# Intimate partner and family violence and mental health during the SARS-CoV-2 pandemic: a multi-country survey

**DOI:** 10.3389/fpsyt.2025.1539075

**Published:** 2025-06-23

**Authors:** Roxanne Keynejad, Oliva Nalwadda, Bushra Syed, Buruwaa Adomako Agyekum, Razia Sultana, Mathew Mutiso, Vishal Bhavsar

**Affiliations:** ^1^ Section of Women’s Mental Health, King’s College London, London, United Kingdom; ^2^ South London and Maudsley National Health Service (NHS) Foundation Trust, London, United Kingdom; ^3^ Uzima Ari, Kampala, Uganda; ^4^ Department of Psychiatry, Government Medical College, Srinagar, India; ^5^ Department of Psychology, University of Ghana, Accra, Ghana; ^6^ Division of Psychology & Mental Health, University of Manchester, Manchester, United Kingdom; ^7^ Coalition Action for Preventive Mental Health, Nairobi, Kenya

**Keywords:** intimate partner violence, family violence, COVID-19, common mental disorders, global mental health, surveys

## Abstract

**Background:**

During COVID-19, concerns were raised about a 'shadow pandemic' of violence against women and girls. However, UN guidance discouraged direct enquiry about intimate partner and family violence (IPFV), instead advocating proxy questions on subjects like relationship difficulties and perceived safety. We investigated the relationship between partner difficulties and family difficulties and common mental disorder (CMDs) during COVID-19 in low-, middle-, and high-income countries.

**Methods:**

We carried out and analysed an online survey, measuring partner difficulties and family difficulties (as proxy items for IPFV), CMDs, and socioeconomic risk factors.

**Results:**

There were 409 respondents in 19 countries. The prevalence of CMDs was 32.27%. After all adjustments, the risk ratio (RR) for the association of partner difficulties with CMD was 1.30 (95% CI: 1.06, 1.60). The adjusted RR of family difficulties with CMD was 1.18 (95% CI: 0.97, 1.44). Both partner and family difficulties were significantly associated with CMD in women [partner difficulties RR = 1.31 (95% CI: 1.05, 1.65); family difficulties RR = 1.37 (95% CI: 1.09, 1.72)].

**Conclusion:**

Collecting proxy data on IPFV is feasible and was related to CMDs during COVID-19 in a range of settings. Like partner violence, family violence may also be related to increased CMDs, especially in women. Policy responses for post-pandemic recovery and preparation for future emergencies should consider the health impacts of family violence as well as partner violence.

## Introduction

Violence towards intimate partners and family members [intimate partner and family violence (IPFV)] are common and related forms of interpersonal violence (1), often rooted in gender inequity and associated with a range of adverse health outcomes (2). Most (3–7) but not all (8–12) research suggests that intimate partner violence (IPV) increased during the COVID-19 pandemic; far fewer studies measured violence perpetrated by non-partner family members (13). For example, a review of 29 studies of family violence during the pandemic included mainly studies of violence towards children, with five included studies where IPV and family violence were grouped, complicating the interpretation of these findings in terms of non-partner family violence (14). A recent systematic review of IPV prevalence during the COVID-19 pandemic focused on women only and identified 14 studies (15). Understanding patterns of IPFV in different contexts during the coronavirus pandemic is important for societal restitution (16), public health and socioeconomic recovery (17), and planning for future emergencies.

A range of evidence supports bidirectional associations between IPV and adverse mental health, including alcohol use disorders (18), depressive symptoms, and suicide attempts (19). Post-traumatic stress disorder, anxiety and depressive disorders (20), and use of secondary mental health services (21) are all known to be associated with IPV exposure. However, although the impact of pandemic restrictions on women’s mental health was widely researched, measurement of IPFV exposure was markedly absent from many studies (22).

There is evidence that both the risk of IPFV exposure and mental health conditions are increased by socioeconomic conditions, including lower educational attainment, unemployment, over-crowded living situations, and more limited social support (23–25).

Whether IPFV increased the risk of common mental disorders (CMD: depression and anxiety disorders) during COVID-19 has been considered by several studies (4, 26, 27). However, interpreting this evidence poses various challenges. Despite the relevance of family violence to pandemic restrictions (28), of the few studies examining family violence during the pandemic, even fewer considered its impact on mental health (13, 29). Furthermore, studies examining IPFV during the pandemic come overwhelmingly from high-income countries (HICs), whose context, pandemic response, and COVID-19 mortality and morbidity rates differed substantially from those of many low- and middle-income countries (LMICs). Also, nearly all survey studies of IPFV during the pandemic asked respondents directly about their experience of IPFV victimisation. This approach has been discouraged by international agencies due to the potential risks of harm to participants disclosing abuse where perpetrators may be present (30). The possibility of under-reporting and non-response due to social stigma and participants’ safety concerns has also been raised (31).

Accordingly, we did a survey using two proxy variables for IPFV (self-reported difficulties in intimate partner and family relationships) to investigate the impact of IPFV on CMDs during the COVID-19 pandemic in low-, middle-, and high-income countries. We aimed to estimate associations of these proxy variables with CMDs and to explore moderation by gender and continent of residence.

## Methods

### Data collection

We carried out a survey among adults aged 16 years and above who were able to read in English, had access to the internet, and had access to a device with which to complete the survey. We invited participation through Twitter, email, and word of mouth and administered the survey through the Qualtrics online platform. Individuals were encouraged not to participate unless they were able to do so in a private place where they would not be disturbed or their answers would not be read by another person. There was no upper age limit. Based on study group members' locations, eight countries were targeted for recruitment: the United Kingdom, Bangladesh, India, Zambia, Ethiopia, South Africa, Kenya, and Uganda. However, people living in any country were eligible to participate. The survey website presented prospective participants with details of support organisations for IPFV and mental health before the survey questions. In Kenya, trained data collectors verbally informed eligible community members about the survey and offered participants a mobile device, if they did not have one, on which to complete the survey. The full survey including accompanying information and item wordings is included in the **Supplementary Material**.

### Ethical approval

Ethical approval was provided by the King’s College London Psychiatry, Nursing and Midwifery research ethics subcommittee (reference: HR-19/20-19295).

### Measurement

#### Partner difficulties and family difficulties

In the context of the United Nations guidance not to inquire directly about IPFV victimisation in online COVID-19 research (30), we developed a set of proxy questions, framed around relationship difficulties with different people before and during the COVID-19 outbreak.

#### Relationship difficulties

A series of items on relationship difficulties assessed difficulties in the following relationship domains: intimate partners, non-partner family members, neighbours, and friends. For each relationship domain, respondents were asked about the degree of difficulties: few, some, moderate, or severe difficulties. For each item, respondents were also asked if these difficulties had worsened since pandemic restrictions began, or had, got better, or not changed. We used difficulties with friends and neighbours to derive an “other relationship difficulties” variable, in comparison to difficulties with partners and family members. We derived variables for increased partner difficulties and increased family difficulties, based on endorsement of whether each respective set of difficulties had improved, stayed the same, or worsened since the pandemic began.

#### Common mental disorders

We used the 20-item WHO Self-Report Questionnaire (SRQ-20) (32) to capture symptoms of CMDs, including depression and anxiety disorders. The SRQ-20 was developed for the screening of CMDs in primary care settings and displays adequate psychometric performance (33). To operationalise the presence of CMDs, we applied a cut-off, such that scoring 7 on the SRQ-20 indicated no CMD and 8 indicated the presence of CMD, in line with previous work (34). The analysis of this dichotomous outcome using Poisson regression with robust standard errors is described below. We also analysed the SRQ-20 score as a continuous dependent variable to supplement the main analysis.

#### Other variables

We collected self-reported demographic information on respondents’ age, gender, country of residence, employment status (student, employed, self-employed, unemployed, full-time carer, and other), and highest educational attainment (primary, secondary, undergraduate, and postgraduate level). Country of residence data were grouped into continents: Asia, the Americas (combining respondents from Brazil and the USA), Europe, and Africa. Using items for the number of people in the household and the number of rooms, we derived a variable for the number of persons per room in the household. We measured perceived social support using items from the third Oslo Social Support Scale (OSSS-3) (35), summed to generate a continuous variable. The continuous measure was based on the total score on three Likert scale-scored items, the number of people one can call upon during personal problems: interest and concern shown by others, and ease of access to practical help from friends and neighbours.

### Analysis

We analysed data using Stata 17 (36). We reported the prevalence of CMDs, and partner and family relationship difficulties by all covariates, using counts and proportions. To describe the data (see **Table 1**), we dichotomised partner and family difficulties into few/some difficulties and moderate/severe difficulties—we handled these variables as continuous variables for the modelling described below. We dichotomised all items on pandemic-related change into no change/improved and worsened, and we dichotomised perceived social support into low and high, at the median, which was 10 (see **Table 1**).

**Table 1:** Description of sample.

	N in row category	CMD^a^, N (%)	Partner difficulties^b^, N (%)	Increased partner difficulties^c^, N (%)	Family difficulties^d^, N (%)	Increased family difficulties^e^, N (%)
Age bands						
17–28	204	72 (35.29)	15 (7.35)	23 (11.27)	28 (13.73)	34 (16.67)
29–40	162	49 (30.25)	25 (15.43)	40 (24.69)	16 (9.88)	29 (17.90)
41–52	22	4 (18.18)	2 (9.09)	9 (36.36)	0 (0.00)	5 (22.73)
53–65	12	3 (25.00)	0 (0.00)	2 (16.67)	0 (0.00)	2 (16.67)
Missing	9	4 (44.44)	1 (0.21)	2 (0.41)	1 (0.21)	2 (0.41)
Gender						
Men	136	43 (31.62)	13 (9.56)	20 (14.71)	9 (6.62)	12 (8.82)
Women	264	86 (32.58)	30 (11.36)	54 (20.45)	35 (13.26)	58 (21.97)
Other	1	0 (0.00)	0 (0.00)	0 (0.00)	0 (0.00)	0 (0.00)
Missing	8	3 (37.50)	0 (0.00)	1 (0.21)	1 (0.21)	2 (0.41)
Continent^f^						
Asia	126	51 (40.16)	11 (8.66)	18 (14.17)	15 (11.81)	25 (19.69)
The Americas	6	2 (33.33)	1 (16.67)	2 (33.33)	2 (33.33)	4 (66.67)
Europe	65	30 (46.15)	9 (13.85)	26 (40.00)	3 (4.62)	15 (23.08)
Africa	203	45 (22.17)	21 (10.34)	28 (13.79)	24 (11.82)	24 (11.82)
Missing	8	4 (50.00)	1 (0.21)	1 (0.21)	1 (0.21)	4 (0.83)
Educational attainment						
Secondary school	53	10 (18.87)	5 (9.43)	6 (11.32)	9 (16.98)	7 (13.21)
Undergraduate degree	154	50 (32.47)	15 (9.74)	28 (18.18)	18 (11.69)	28 (18.18)
Postgraduate degree	192	66 (34.38)	21 (10.94)	38 (19.79)	15 (7.81)	33 (17.19)
Missing	10	6 (60.00)	2 (0.41)	3 (0.62)	3 (0.62)	4 (0.82)
Employment						
Student	128	42 (32.81)	10 (7.81)	12 (9.38)	21 (16.41)	19 (14.84)
Employed	163	52 (31.90)	19 (11.66)	42 (25.77)	11 (6.75)	33 (20.25)
Self-employed	53	12 (22.64)	4 (7.55)	7 (13.21)	3 (5.66)	5 (9.43)
Unemployed	32	16 (50.00)	6 (19.75)	9 (28.12)	5 (15.62)	8 (25.00)
Full-time carer	2	0 (0.00)	0 (0.00)	0 (0.00)	0 (0.00)	0 (0.00)
Other	21	5 (23.81)	2 (9.52)	4 (19.05)	2 (9.52)	5 (23.81)
Missing	10	5 (50.00)	2 (0.41)	1 (0.21)	3 (0.62)	2 (0.41)
Persons per room^g^						
Less than 1	191	63 (32.98)	15 (7.89)	39 (20.63)	17 (8.99)	28 (14.81)
1–2	158	50 (31.65)	18 (11.32)	24 (15.00)	19 (11.88)	29 (18.12)
2 or more	45	15 (33.33)	8 (17.39)	9 (20.00)	8 (17.78)	13 (28.89)
Missing	15	4 (26.67)	2 (0.41)	3 (0.61)	1 (0.20)	2 (0.41)
Perceived social support^h^						
Low support	249	101 (40.56)	38 (15.26)	55 (22.09)	39 (15.66)	54 (21.69)
High support	143	27 (18.88)	5 (3.50)	20 (13.99)	6 (4.20)	18 (12.59)
Missing	17	4 (23.53)	0 (0.00)	0 (0.00)	0 (0.00)	0 (0.00)
Other relationship difficulties^i^						
No	248	59 (23.71)	20 (8.06)	45 (18.15)	13 (5.24)	35 (14.11)
Yes	141	70 (49.65)	23 (16.31)	30 (21.28)	32 (22.70)	37 (26.24)
Missing	20	3 (15.00)	0 (0.00)	0 (0.00)	0 (0.00)	0 (0.00)
**Overall**	**409**	**132 (32.27)**	**43 (10.51)**	**75 (18.34)**	**45 (11.00)**	**72 (17.60)**

a CMD, common mental disorder. CMD was operationalised as a score of 8 or more on the WHO self-reporting questionnaire (SRQ-20).

b Defined as reporting moderate or severe difficulties on the question “How many difficulties are you experiencing with your romantic or marital partner/spouse at the moment?”.

c Defined as reporting that difficulties had worsened since the national government’s response to the pandemic began.

d Defined as reporting moderate or severe difficulties to the question “How many difficulties are you experiencing in your relationship with other family members (non-partners/spouses)?”.

e Defined as reporting that difficulties had worsened since the national government’s response to the pandemic began.

f Based on self-reported country of residence.

g Based on responses to questions about the number of children and adults living in the household and number of rooms in the household.

h For description, social support was measured as a continuous variable and then dichotomised at the median (10). The continuous measure was based on the total score on three items: “How many people are so close to you that you can count on them if you have great personal problems?”, with possible responses of none, 1–2, 3–5, and more than 5; “How much interest and concern do people show in what you do?” (none, little, uncertain, some, or a lot); and “How easy is it to get practical help from friends and neighbours if you should need it?” (very difficult, difficult, possible, easy, or very easy). For modelling, social support was included in models as a continuous variable.

i Defined as reporting moderate or severe difficulties in relationships with work colleagues, and friends.

As described under the Measurement section, the outcome for analysis was (dichotomous) CMD. Assumptions of odds ratios as measures of relative risk may be violated where the outcome is very common (e.g. greater than 10%). Given the high prevalence of CMDs in our data, we used robust (or modified) Poisson regression to model the outcome, as recommended by Zou (37). To evaluate the association of partner difficulties and family difficulties with CMD, we used robust Poisson regressions estimating the risk ratio (RR) for the association of a unit change in each exposure variable with CMD. After inspecting unadjusted estimates including only the exposures and CMD (model I), we included basic sociodemographic variables (age, gender, and continent of residence: model II). We then included socioeconomic and social support variables (employment status, educational attainment, persons per household room, and perceived social support score: model III) and then pandemic-related change (model IV). Finally, we included general difficulties for each exposure (model V): for partner relationship difficulties, we adjusted for non-partner relationship difficulties; for family relationship difficulties, we adjusted for non-family relationship difficulties; and for home safety, we adjusted for perceived safety outside the home environment. Lastly, to evaluate associations stratified by gender and continent of residence, we estimated models including a multiplicative interaction term for each of these variables. We produced post-estimation fitted estimates for the association in each group and tested for significant heterogeneity using likelihood ratio tests.

## Results

The survey received 409 responses (264 female, 136 male, one other gender, and eight for whom gender was missing) from individuals living in 19 countries (Bangladesh, Brazil, Denmark, Germany, Ghana, Greece, India, Italy, Kenya, Malta, Singapore, South Africa, Sri Lanka, Uganda, the United Arab Emirates, the United Kingdom, the USA, Zambia, and Zimbabwe) on four continental regions (Asia, the Americas, Europe, and Africa).

The prevalence of CMD was 32.27% (see **Table 1**). The prevalence of partner difficulties was 10.51%, and 18.34% of participants reported worsening partner difficulties since the pandemic began. The proportion of participants reporting family relationship difficulties was 11.00%, and 17.60% reported worsening family difficulties since the beginning of the pandemic.

### CMD and covariates

The highest prevalence of CMD was in Europe (46.15%), then Asia (40.16%), the Americas (33.33%), and Africa (22.17%, see **Table 1**). The prevalence of CMDs among those with low perceived social support was 40.56%, and among those with high social support, it was 18.88%. Among those reporting difficulties in non-partner or non-family relationships, CMD prevalence was 49.65%, and in those not reporting these difficulties, it was 23.71%.

### Partner difficulties and family difficulties

#### Partner difficulties

Respondents in the 29–40 years of age category had a CMD prevalence of 24.69%, and in the 41–52 years of age category, it was 36.36%. The prevalence of CMDs in the youngest age category (17–28) was 11.27%, and in the oldest age category (53–65), it was 16.67%. The proportion of respondents in Europe who reported worsening partner difficulties was 40%, while in Asia, it was 14.17%. In those with low perceived social support, the prevalence of partner difficulties was 15.26%, and among those with high social support, it was 3.50%. The prevalence of reporting worsening partner difficulties since the pandemic began was 25.77% among those who were employed, 28.12% among those unemployed, and 9.38% among students.

#### Family difficulties

The proportion of women who reported worsening family difficulties since the pandemic began was 21.97%, and the proportion of men doing so was 8.82%. Family difficulties had a prevalence of 15.66% among those with low social support and 4.20% among those with high social support. The prevalence of worsening family difficulties since the pandemic began was 14.81% in those with <1 person per room and 28.89% in those with 1–2 persons per room.

### The association of partner difficulties and family difficulties with CMD

In the unadjusted model, there was an association of greater partner difficulties with CMD (RR = 1.50, 95% CI: 1.30, 1.72, p < 0.001, **Table 2**). In comparison, the association of greater family difficulties with CMD was closer to null but remained statistically significant (RR = 1.22, 95% CI: 1.04, 1.42, p = 0.012). The association of family difficulties with CMD remained similar after all adjustments but was no longer statistically significant (RR = 1.18, 95% CI: 0.97, 1.44, p = 0.89). There was some attenuation of the association of partner difficulties with CMD after all adjustments to RR = 1.30 (95% CI: 1.06, 1.60, p = 0.01).

**Table 2:** Estimates for the association [in the form of risk ratios (RRs) with 95% confidence intervals in brackets and p-values with statistically significant estimates in bold] of partner difficulties, and family difficulties.

	Partner/family difficulties	Model I (277 observations)	Model II (264 observations)	Model III (254 observations)	Model IV (254 observations)	Model V (253 observations)
Overall	Family difficulties	**1.22 (1.04, 1.42),** **0.012**	**1.20 (1.02, 1.42),** **0.029**	1.18 (0.97, 1.43), 0.080	1.17 (0.96, 1.43), 0.106	1.18 (0.97, 1.44), 0.89
	Partner difficulties	**1.50 (1.30, 1.72), <0.001**	**1.48 (1.28, 1.71),** **<0.001**	**1.34 (1.21, 1.61),** **0.001**	**1.31 (1.07, 1.61),** **0.006**	**1.30 (1.06, 1.60),** **0.01**
Men	Family difficulties	1.11 (0.82, 1.50), 0.515	1.05 (0.74, 1.48), 0.797	0.93 (0.61, 1.42), 0.852	0.93 (0.61, 1.43), 0.839	0.95 (0.63, 1.44), 0.888
	Partner difficulties	**1.31 (1.01, 1.69),** **0.04**	1.24 (0.88, 1.73), 0.066	1.22 (0.89, 1.66), 0.268	1.20 (0.86, 1.68), 0.303	1.17 (0.83, 1.66), 0.436
Women	Family difficulties	**1.27 (1.06, 1.52),** **0.009**	**1.26 (1.05, 1.52),** **0.014**	**1.33 (1.08, 1.64),** **0.009**	**1.31 (1.06, 1.64),** **0.014**	**1.31 (1.05, 1.65),** **0.023**
	Partner difficulties	**1.60 (1.35, 1.91),** **<0.001**	**1.60 (1.33, 1.92),** **<0.001**	**1.39 (1.11, 1.73),** **0.002**	**1.37 (1.09, 1.73),** **0.003**	**1.37 (1.09, 1.72),** **0.003**
Asia	Family difficulties	1.10 (0.84, 1.42), 0.490	1.09 (0.83, 1.44), 0.533	1.19 (0.78, 1.82), 0.457	1.18 (0.78, 1.77), 0.470	1.21 (0.78, 1.89), 0.483
	Partner difficulties	**1.42 (1.14, 1.78),** **0.002**	**1.40 (1.10, 1.78),** **0.006**	1.09 (0.74, 1.60), 0.581	1.07 (0.73, 1.56), 0.616	1.01 (0.67, 1.51), 0.835
America	Family difficulties	1.44 (0.85, 2.46), 0.179	1.44 (0.84, 2.45), 0.187	1.45 (0.86, 2.44), 0.204	1.40 (0.81, 2.42), 0.275	1.39 (0.80, 2.41), 0.300
	Partner difficulties	–	–	–	–	–
Europe	Family difficulties	**1.25 (1.02, 1.53),** **0.033**	1.18 (0.95, 1.47), 0.128	1.16 (0.91, 1.47), 0.215	1.11 (0.83, 1.49), 0.416	1.11 (0.82, 1.51), 0.392
	Partner difficulties	**1.44 (1.20, 1.74),** **<0.001**	**1.45 (1.18, 1.79),** **<0.001**	**1.38 (1.08, 1.77),** **0.006**	**1.36 (1.02, 1.82),** **0.022**	**1.34 (1.01, 1.78),** **0.033**
Africa	Family difficulties	1.29 (0.98, 1.70), 0.066	1.26 (0.96, 1.66), 0.093	1.25 (0.92, 1.70), 0.096	1.22 (0.89, 1.67), 0.142	1.21 (0.89, 1.66), 0.154
	Partner difficulties	**1.70 (1.29, 2.23),** **<0.001**	**1.71 (1.30, 2.24),** **<0.001**	**1.59 (1.16, 2.17),** **0.005**	**1.59 (1.17, 2.17),** **0.005**	**1.55 (1.14, 2.10),** **0.005**

Model I included no other variables and estimated the “crude” association. Model II included age, gender, and continent. Model III included model II variables and added social support, persons per room, employment status, and educational attainment. Model IV included model III variables and added worsened partner difficulties and worsened family difficulties during the pandemic. Model V included model IV variables and added other relationship difficulties. Interaction term for gender and family difficulties: z = 1.22, p = 0.224. Interaction term for gender and partner difficulties: z = 1.09, p = 0.276. Interaction term for continent and family difficulties: X^2^ = 1.01, 3 degrees of freedom, p = 0.7988. Interaction term for continent and partner difficulties: X^2^ = 17.06, 3 degrees of freedom, p = 0.007. “-” indicates that models’ estimates for these categories were not produced due to small numbers of participants in the corresponding strata. Bold indicates statistically significant associations.

On stratifying by gender, in the final models, both partner and family difficulties were significantly associated with CMD in women [partner difficulties: RR = 1.37 (95% CI: 1.09, 1.72, p = 0.003); family difficulties: RR = 1.31 (95% CI: 1.05, 1.65, p = 0.023)]. However, in men, the estimates did not reach statistical significance and were closer to null. We found no evidence for significant heterogeneity in the association of either partner or family difficulties with CMD between men and women.

On stratifying by continent, there was statistical evidence for the association of partner difficulties with CMD in Europe [RR = 1.34 (95% CI: 1.01, 1.78, p = 0.033)] and Africa (RR = 1.55, 95% CI: 1.14, 2.10, p = 0.005), but not in Asia. The estimates for the association of partner difficulties with CMD were non-significant and very close to null for Asia (RR = 1.01, 95% CI: 0.67, 1.51) and statistically significant for Europe (RR = 1.34, 95% CI: 1.01, 1.78) and Africa (RR = 1.55, 95% CI: 1.14, 2.10), while estimates were not produced for the Americas owing to small numbers of respondents in this group. There was statistical evidence for interaction by continent in the association of partner difficulties with CMD (p = 0.007), but not of family difficulties with CMD (p = 0.7988).

## Discussion

### Summary of findings

Self-reported partner difficulties and family difficulties were associated with CMDs after accounting for possible confounders, including pandemic-related change in each exposure variable. Estimates for the association of partner difficulties and family difficulties, with CMD were stronger in women than in men, but we did not find statistical evidence for interaction by gender. While there was statistical evidence for interaction in the association of partner difficulties with CMD by continent, this was based on small numbers of respondents from the Americas, so this result should be treated with caution.

### Interpretation

Our results suggest that partner difficulties and family difficulties are associated with CMD and are not attributable to differences in individual sociodemographic characteristics, socioeconomic status, or perceived social support. Point estimates suggested that the relationship between family difficulties and CMDs may be somewhat weaker in men than women, although we did not find statistical evidence for interaction, and this requires further study. Across the whole sample, the relationship of partner difficulties and family difficulties with CMDs appeared to persist, irrespective of the experience of change in difficulties since the beginning of the pandemic. Based on our analysis, the association of partner difficulties and family difficulties with CMD is unlikely to be due to the non-specificity of the markers themselves. That is, it is unlikely that the relationship between partner difficulties and CMDs is explained by difficulties in wider relationships (family, friends, and neighbours), and it is unlikely that the relationship between family difficulties and CMDs is explained by difficulties in wider relationships (partner, friends, and neighbours), as we adjusted for these factors in our analyses. This adds confidence that our estimates reflect the impact of difficulties in each specific relationship domain, rather than non-partner or family-related factors, which may result in endorsing these items.

We found that partner difficulties and family difficulties were associated with CMDs across diverse geographical settings, including in LMICs, which largely enforced less restrictive lockdowns than HICs (38–41). This could indicate that the degree of restrictions at a policy level had a limited impact on the mental health impact of IPV in contrast to other factors such as economic insecurity or fears of infection, which may have varied less across countries, or that the effect of restrictions was partly explained by other factors, such as local variation in compliance with these restrictions (42). Our finding of a stronger association of partner difficulties with CMDs in Europe and Africa, compared to Asia, warrants further research in large representative samples.

Our results are consistent with the effect of partner difficulties and family difficulties on CMDs during the pandemic. Mechanisms could include prolonged anxiety and psychosocial stress (for example, about finances and livelihoods) and psychological mechanisms reflecting a sense of entrapment in abusive relationships. Stronger point estimates for the association of family difficulties with CMDs in women compared to men are in line with previous research suggesting that family demands have more damaging effects on women’s mental health, compared to men (43, 44), but again, we did not find statistical evidence for interaction, and this hypothesis should be tested in future studies.

### How our study fits with previous literature

The collection of research data on IPFV during the pandemic shifted in large part to remote methods (45). Following international ethical guidance, we employed self-reported partner difficulties, family difficulties, and perceived home safety as proxy markers for IPFV. Several studies have demonstrated a relationship between (directly measured) IPV and CMD during the pandemic, although many were conducted in HICs (4, 8, 26, 27, 46–49). There have been limited attempts to consider the impact of pre-pandemic IPV on CMDs during COVID-19. By accounting for pre-pandemic IPV models, we increase confidence that associations of partner difficulties and family difficulties with CMD are not attributable to pre-pandemic difficulties in relationships.

Although some studies reported increased family violence (FV) during the pandemic (50, 51), we present the first evidence (to our knowledge) that family relationship difficulties are associated with CMDs in a pandemic context. Previous research found that dissatisfaction with family relationships was strongly associated with psychological distress (scoring moderate or high on the 10-item Kessler Psychological Distress Scale) in New Zealand during the pandemic (42). Although this study also measured the experience of family violence, associations of family violence with distress were not presented.

A limited number of studies has examined the mental health impact of partner difficulties in the pandemic context. Although partner difficulties are associated with CMDs (19, 20, 52–54), evidence for this relationship during COVID-19 is limited, despite emerging evidence on the prevalence of IPV during this period (14, 15).

### Limitations

Our analysis of this cross-sectional survey cannot determine the temporal relationship of partner difficulties or family difficulties with CMDs. Our sample was not representative of all people in the countries sampled, and respondents with access to devices, the internet, and social media are likely to represent a source population with higher levels of educational attainment and socioeconomic status. Both factors influence the risk of IPFV and CMD, as well as the likelihood of adverse impacts of pandemic restrictions. Our findings may therefore under-estimate the prevalence of both IPFV and CMDs. Survey response was variable between continents surveyed—for example, a small number of respondents in the Americas rendered association estimates for partner difficulties in this region unstable. We measured CMDs using an instrument designed to define community prevalence, and our results may not generalise to the association of IPFV with clinically ascertained depression. While variation in the prevalence of CMDs, for example, a greater prevalence in Europe compared to Africa, ascertained in our study is consistent with other studies carried out during the pandemic (55), we cannot discount the possibility of variable under-ascertainment of CMDs across geographical settings, which would introduce bias.

Items measuring partner and family difficulties did not specify forms of IPFV (physical, sexual, and emotional) in detail to safeguard respondents. We were therefore unable to consider the correspondence of our exposure data with direct self-reported measures of IPFV, such as those collected using the Conflict Tactics Scales and other measures (56). We also did not measure coercive control, so we could not disaggregate the presence of coercive control within the group reporting partner difficulties or family difficulties. The relationship that we identified between proxies for IPFV and CMDs during the pandemic may have been confounded by the availability of mental health support and services, which we did not measure and which varies between geographical settings (19). In this paper, we employed partner and family relationship difficulties as indirect indicators of IPFV. Although meta-analytic evidence suggests that self-reported relationship satisfaction is strongly negatively correlated with partner violence (57), this should be considered when generalising our results from partner and family difficulties to IPFV.

## Implications and conclusions

We found that both partner and family difficulties were associated with CMDs in a recent peri-pandemic sample. Point estimates for these associations were greater in magnitude in women than men, especially for family difficulties. Measures to identify and safely provide evidence-based support for people experiencing relationship difficulties during the pandemic may be beneficial for mental health.

## Data Availability

The datasets presented in this article are not readily available because they contain information that could compromise the privacy of research participants. Requests to access the datasets should be directed to vishal.2.bhavsar@kcl.ac.uk.

## References

[1] Krug E Dahlberg L Mercy J Zwi A Lozano R. World health report on violence and health. Geneva: World Health Organization (2002).

[2] Oram S Fisher HL Minnis H Seedat S Walby S Hegarty K. The Lancet Psychiatry Commission on intimate partner violence and mental health: advancing mental health services, research, and policy. Lancet Psychiatry. (2022) 9:487–524. doi: 10.1016/S2215-0366(22)00008-6 35569504

[3] Jetelina KK Knell G Molsberry RJ. Changes in intimate partner violence during the early stages of the COVID-19 pandemic in the USA. Injury Prev. (2021) 27:93–7. doi: 10.1136/injuryprev-2020-043831 32873603

[4] Ebert C Steinert JI. Prevalence and risk factors of violence against women and children during COVID-19, Germany. Bull World Health Organization. (2021) 99:429. doi: 10.2471/BLT.20.270983 PMC8164185 34108753

[5] Decker MR Bevilacqua K Wood SN Ngare GW Thiongo M Byrne ME. Gender-based violence during COVID-19 among adolescent girls and young women in Nairobi, Kenya: a mixed-methods prospective study over 18 months. BMJ Global Health. (2022) 7. doi: 10.1136/bmjgh-2021-007807 PMC8882641 35210310

[6] Schokkenbroek JM Anrijs S Ponnet K Hardyns W. Locked down together: determinants of verbal partner violence during the COVID-19 pandemic. Violence gender. (2021) 8:148–53. doi: 10.1089/vio.2020.0064

[7] Nesset MB Gudde CB Mentzoni GE Palmstierna T. Intimate partner violence during COVID-19 lockdown in Norway: the increase of police reports. BMC Public Health. (2021) 21:1–8. doi: 10.1186/s12889-021-12408-x 34915874 PMC8677344

[8] Ojeahere MI Kumswa SK Adiukwu F Plang JP Taiwo YF. Intimate partner violence and its mental health implications amid COVID-19 lockdown: findings among Nigerian couples. J interpersonal violence. (2022) 37:NP15434–NP54. doi: 10.1177/08862605211015213 33993788

[9] Alharbi FF Alkheraiji MA Aljumah AA Al-Eissa M Qasim SS Alaqeel MK. Domestic violence against married women during the COVID-19 quarantine in Saudi Arabia. Cureus. (2021) 13. doi: 10.7759/cureus.15231 PMC8232927 34188981

[10] Kliem S Baier D Kröger C. Domestic violence before and during the COVID-19 pandemic: A comparison of two representative population surveys. Deutsches Arzteblatt Int. (2021) 118:483. doi: 10.3238/arztebl.m2021.0267 PMC8456436 34491161

[11] O’Hara CA Tan RKJ. Intimate partner violence before and during the COVID-19 lockdown: findings from a cross-sectional study in Singapore. Sexual Health. (2022) 19:192–201. doi: 10.1071/SH21229 35636747

[12] Piquero AR Riddell JR Bishopp SA Narvey C Reid JA Piquero NL. Staying home, staying safe? A short-term analysis of COVID-19 on Dallas domestic violence. Am J criminal justice. (2020) 45:601–35. doi: 10.1007/s12103-020-09531-7 PMC7293590 32837161

[13] Augusti E-M Sætren SS Hafstad GS. Violence and abuse experiences and associated risk factors during the COVID-19 outbreak in a population-based sample of Norwegian adolescents. Child Abuse neglect. (2021) 118:105156. doi: 10.1016/j.chiabu.2021.105156 34139385 PMC9757861

[14] Letourneau N Luis MA Kurbatfinski S Ferrara HJ Pohl C Marabotti F. COVID-19 and family violence: A rapid review of literature published up to 1 year after the pandemic declaration. EClinicalMedicine. (2022) 53. doi: 10.1016/j.eclinm.2022.101634 PMC9472575 36119559

[15] Kifle ME Aychiluhm SB Anbesu EW. Global prevalence of intimate partner violence during the COVID-19 pandemic among women: systematic review and meta-analysis. BMC women’s Health. (2024) 24:127. doi: 10.1186/s12905-023-02845-8 38368323 PMC10874578

[16] UN Women. Measuring the shadow pandemic: violence against women during COVID-19. (2021). Available at: https://data.unwomen.org/sites/default/files/documents/Publications/Measuring-shadow-pandemic.pdf.

[17] Sharma A Borah SB. Covid-19 and domestic violence: an indirect path to social and economic crisis. J Family violence. (2022) 37:759–65. doi: 10.1007/s10896-020-00188-8 PMC7386835 32836737

[18] Devries KM Child JC Bacchus LJ Mak J Falder G Graham K. Intimate partner violence victimization and alcohol consumption in women: A systematic review and meta-analysis. Addiction. (2014) 109:379–91. doi: 10.1111/add.2014.109.issue-3 24329907

[19] Devries KM Mak JY Bacchus LJ Child JC Falder G Petzold M. Intimate partner violence and incident depressive symptoms and suicide attempts: a systematic review of longitudinal studies. PloS Med. (2013) 10:e1001439. doi: 10.1371/journal.pmed.1001439 23671407 PMC3646718

[20] Trevillion K Oram S Feder G Howard LM. Experiences of domestic violence and mental disorders: a systematic review and meta-analysis. PloS One. (2012) 7:e51740. doi: 10.1371/journal.pone.0051740 23300562 PMC3530507

[21] Khalifeh H Johnson S Howard LM Borschmann R Osborn D Dean K. Violent and non-violent crime against adults with severe mental illness. Br J Psychiatry. (2015) 206:275–82. doi: 10.1192/bjp.bp.114.147843 25698767

[22] Keynejad RC. Domestic violence and mental health during COVID-19. Prog Neurol Psychiatry. (2023) 27:50–5. doi: 10.1002/pnp.v27.1

[23] Capaldi DM Knoble NB Shortt JW Kim HK. A systematic review of risk factors for intimate partner violence. Partner Abuse. (2012) 3:231–80. doi: 10.1891/1946-6560.3.2.231 PMC3384540 22754606

[24] Lund C Breen A Flisher AJ Kakuma R Corrigall J Joska JA. Poverty and common mental disorders in low and middle income countries: A systematic review. Soc Sci Med. (2010) 71:517–28. doi: 10.1016/j.socscimed.2010.04.027 PMC4991761 20621748

[25] Ehsan AM De Silva MJ. Social capital and common mental disorder: a systematic review. J Epidemiol Community Health. (2015) 69:1021–8. doi: 10.1136/jech-2015-205868 26179447

[26] Abrahams Z Boisits S Schneider M Prince M Lund C. The relationship between common mental disorders (CMDs), food insecurity and domestic violence in pregnant women during the COVID-19 lockdown in Cape Town, South Africa. Soc Psychiatry Psychiatr Epidemiol. (2022) 57:37–46. doi: 10.1007/s00127-021-02140-7 34282488 PMC8288830

[27] Lee SJ Ward KP Rodriguez CM. Longitudinal analysis of short-term changes in relationship conflict during COVID-19: A risk and resilience perspective. J interpersonal violence. (2022) 37:NP14239–NP61. doi: 10.1177/08862605211006359 33866855

[28] Usher K Bradbury Jones C Bhullar N Durkin DJ Gyamfi N Fatema SR. COVID-19 and family violence: Is this a perfect storm? Int J Ment Health Nurs. (2021) 30:1022–32. doi: 10.1111/inm.12876 PMC8242793 34008291

[29] Niederkrotenthaler T Laido Z Kirchner S Braun M Metzler H Waldhör T. Mental health over nine months during the SARS-CoV2 pandemic: representative cross-sectional survey in twelve waves between April and December 2020 in Austria. J Affect Disord. (2022) 296:49–58. doi: 10.1016/j.jad.2021.08.153 34587549 PMC8426850

[30] UN Women. Violence against women and girls data collection during COVID-19. (2020).

[31] Smyth C Cullen P Breckenridge J Cortis N Valentine K. COVID-19 lockdowns, intimate partner violence and coercive control. Aust J Soc issues. (2021) 56:359–73. doi: 10.1002/ajs4.v56.3 PMC8222883 34188336

[32] Beusenberg M Orley JH World Health Organization. “Division of mental health” in A user's guide to the self reporting questionnaire (SRQ/compiled by M. Beusenberg and J. Orley). World Health Organization (1994). Available at: https://iris.who.int/handle/10665/61113.

[33] Ludermir AB Lewis G Valongueiro SA de Araújo TVB Araya R. Violence against women by their intimate partner during pregnancy and postnatal depression: a prospective cohort study. Lancet. (2010) 376:903–10. doi: 10.1016/S0140-6736(10)60887-2 20822809

[34] Ludermir AB Lewis G. Investigating the effect of demographic and socioeconomic variables on misclassification by the SRQ-20 compared with a psychiatric interview. Soc Psychiatry Psychiatr Epidemiol. (2005) 40:36–41. doi: 10.1007/s00127-005-0840-2 15624073

[35] Kocalevent R-D Berg L Beutel ME Hinz A Zenger M Härter M. Social support in the general population: standardization of the Oslo social support scale (OSSS-3). BMC Psychol. (2018) 6:1–8. doi: 10.1186/s40359-018-0249-9 30016997 PMC6050647

[36] StataCorp. Stata 17. StataCorp LP: College Station, TX (2022).

[37] Zou G. A modified poisson regression approach to prospective studies with binary data. Am J Epidemiol. (2004) 159:702–6. doi: 10.1093/aje/kwh090 15033648

[38] Kitano T Kitano M Krueger C Jamal H Al Rawahi H Lee-Krueger R. The differential impact of pediatric COVID-19 between high-income countries and low-and middle-income countries: a systematic review of fatality and ICU admission in children worldwide. PLoS One. (2021) 16:e0246326. doi: 10.1371/journal.pone.0246326 33513204 PMC7845974

[39] Lloyd-Sherlock P Ebrahim S Geffen L McKee M. Bearing the brunt of covid-19: older people in low and middle income countries. Br Med J Publishing Group. (2020) 368:m1052. doi: 10.1136/bmj.m1052 32169830

[40] Gajbhiye RK Sawant MS Kuppusamy P Surve S Pasi A Prusty RK. Differential impact of COVID-19 in pregnant women from high-income countries and low-to middle-income countries: a systematic review and meta-analysis. Int J Gynecol Obstetrics. (2021) 155:48–56. doi: 10.1002/ijgo.v155.1 PMC7612435 34160059

[41] Madhi S A Gray G E Ismail N Izu A Mendelson M Cassim N. COVID-19 lockdowns in low-and middle-income countries: Success against COVID-19 at the price of greater costs. SAMJ: South Afr Med J. (2020) 110:724–6. 32880296

[42] Every-Palmer S Jenkins M Gendall P Hoek J Beaglehole B Bell C. Psychological distress, anxiety, family violence, suicidality, and wellbeing in New Zealand during the COVID-19 lockdown: A cross-sectional study. PLoS One. (2020) 15:e0241658. doi: 10.1371/journal.pone.0241658 33147259 PMC7641386

[43] Artazcoz L Borrell C Benach J. Gender inequalities in health among workers: the relation with family demands. J Epidemiol Community Health. (2001) 55:639–47. doi: 10.1136/jech.55.9.639 PMC1731969 11511642

[44] Marchand A Bilodeau J Demers A Beauregard N Durand P Haines VY III. Gendered depression: Vulnerability or exposure to work and family stressors? Soc Sci Med. (2016) 166:160–8. doi: 10.1016/j.socscimed.2016.08.021 27566045

[45] Amiya B Ellen T Aggrey A Angel M Janet N Jenny P. Remote methods for research on violence against women and children: lessons and challenges from research during the COVID-19 pandemic. BMJ Global Health. (2022) 7:e008460. doi: 10.1136/bmjgh-2022-008460 PMC9676415 36396176

[46] Kimura M Kimura K Ojima T. Relationships between changes due to COVID-19 pandemic and the depressive and anxiety symptoms among mothers of infants and/or preschoolers: a prospective follow-up study from pre-COVID-19 Japan. BMJ Open. (2021) 11:e044826. doi: 10.1136/bmjopen-2020-044826 PMC7907626 33622953

[47] Indu PV Vijayan B Tharayil HM Ayirolimeethal A Vidyadharan V. Domestic violence and psychological problems in married women during COVID-19 pandemic and lockdown: A community-based survey. Asian J Psychiatry. (2021) 64:102812. doi: 10.1016/j.ajp.2021.102812 PMC8514844 34461369

[48] Halperin O Ali-Saleh O Ore L Jadaon JE. Depression, stress and the mediating role of intimate partner violence (IPV) among Israeli women of childbearing age in the shadow of the COVID-19 pandemic. J interpersonal violence. (2023) 38:3586–611. doi: 10.1177/08862605221111415 PMC10076180 35899767

[49] Abrahams Z Lund C. Food insecurity and common mental disorders in perinatal women living in low socio-economic settings in Cape Town, South Africa during the COVID-19 pandemic: a cohort study. Global Ment Health. (2022) 9:49–60. doi: 10.1017/gmh.2022.12 PMC8861552 36606240

[50] Campbell AM. An increasing risk of family violence during the Covid-19 pandemic: Strengthening community collaborations to save lives. Forensic Sci International: Rep. (2020) 2:100089. doi: 10.1016/j.fsir.2020.100089 PMC7152912 38620174

[51] van Gelder N Peterman A Potts A O’Donnell M Thompson K Shah N. COVID-19: Reducing the risk of infection might increase the risk of intimate partner violence. eClinicalMedicine. (2020) 21. doi: 10.1016/j.eclinm.2020.100348 PMC7151425 32292900

[52] Kouros CD Papp LM Cummings EM. Interrelations and moderators of longitudinal links between marital satisfaction and depressive symptoms among couples in established relationships. J Family Psychol. (2008) 22:667–77. doi: 10.1037/0893-3200.22.5.667 18855503

[53] Bulloch AG Williams JV Lavorato DH Patten SB. The relationship between major depression and marital disruption is bidirectional. Depression Anxiety. (2009) 26:1172–7. doi: 10.1002/da.v26:12 19798680

[54] Clavarino A Hayatbakhsh MR Williams GM Bor W O’Callaghan M Najman JM. Depression following marital problems: different impacts on mothers and their children? A 21-year prospective study. Soc Psychiatry Psychiatr Epidemiol. (2011) 46:833–41. doi: 10.1007/s00127-010-0253-8 20574844

[55] Mahmud S Mohsin M Dewan MN Muyeed A. The global prevalence of depression, anxiety, stress, and insomnia among general population during COVID-19 pandemic. A Systematic Rev Meta-analysis. (2023) 31:143–70. doi: 10.21203/rs.3.rs-1136589/v1 PMC8726528 40477944

[56] Straus MA Douglas EM. A short form of the Revised Conflict Tactics Scales, and typologies for severity and mutuality. Violence victims. (2004) 19:507–20. doi: 10.1891/vivi.19.5.507.63686 15844722

[57] Stith SM Smith DB Penn CE Ward DB Tritt D. Intimate partner physical abuse perpetration and victimization risk factors: A meta-analytic review. Aggression Violent Behavior. (2004) 10:65–98. doi: 10.1016/j.avb.2003.09.001

