# Supplementary material for: Intimate partner and family violence and mental health during the SARS-CoV-2 pandemic: a multi-country survey
Source: Front Psychiatry. 2025 Jun 23;16:1539075. doi: 10.3389/fpsyt.2025.1539075 (PMC12230061; doi:10.3389/fpsyt.2025.1539075)
Supplement: Supplementary file 1 [file SupplementaryFile1.docx]

**SUPPLEMENTARY MATERIAL**

**This is a survey about your wellbeing during the Coronavirus pandemic and how the situation has affected you and your relationships.**

- **As the questions are personal, we recommend that you complete them in a private space and do not read them out loud if you can be heard.**
- **Taking part is voluntary and you can stop at any time.**
- **We will not be collecting your name or personal information. Your responses are anonymous and will not be used to identify you.**

**If you are experiencing stress, distress, physical or mental health problems, suicidal thoughts, abuse or violence, you are not alone. Your local health service can help.**

- **If you or someone else is at immediate risk, you can call emergency services for police or ambulance support.**
- **Below are some organisations who provide help, but if your country is not listed, you can find others online or by asking a health worker at your nearest health centre.**

**UGANDA**

- Butabika National Referral Mental Hospital <https://www.butabikahospital.go.ug/> (+256 414 504 376-day time +256 414 671 019 (Evening/Night Shift))
- Mental Health Uganda <https://www.mentalhealthuganda.org/> ([info@mentalhealthuganda.org](mailto:info@mentalhealthuganda.org) +256-392-178-953)
- Strong Minds <https://strongminds.org/> +256 200923340.
- Uganda Women’s Network <https://www.uwonet.or.ug/> (+256759330000)
- ALIGHT UGANDA PROGRAM <https://wearealight.org/> (toll free, 24 hours for refugees and asylum seekers +256771007447)

**ETHIOPIA**

- <http://www.epanets.org/>
- <https://www.womankind.org.uk/what-we-do/our-approach/partners/detail/association-for-women-s-sanctuary-and-development-(awsad)>

**ZAMBIA**

- PsycHealth Zambia https://psychzambia.com/ (+260955264975)
- Psychology Association of Zambia https://paz.co.zm/ (+260762024357)
- Strong Minds Zambia https://strongminds.org/ (+256 200923340)
- University Teaching Hospital, Clinic 6 /Department of Psychiatry https://www.unza.zm/schools/medicine/departments/psychiatry (+260211253947)
- Lifeline Zambia https://clzambia.org/ (933 Toll free) Victim Support Unit Desk available at every police station.

**INDIA**

- National Institute of Mental Health & Neurosciences (NIMHANS): 080 – 4611 0007 (24*7 Toll-Free Helpline)
- iCall Phonelines (Multilingual) +919152987821 (10 am to 8 pm, Monday to Saturday), chat: nULTA app (10:00-20:00, Monday to Saturday), email: [icall@tiss.edu](mailto:icall@tiss.edu)
- Dedicated COVID support helpline: +91 9152987820 (10:00-18:00 Monday to Saturday)
- Institute of Mental Health and Neurosciences, Kashmir (IMHANS-K) Tel: 0194-2410037, Covid-19 mental health helplines: 7006314064 or 6006175374
- IMHANS-K in collaboration with Athrout COVID 19 dedicated mental health helplines: 6006613436, 6006613437 or 6006613438 10:00-13:00 and 16:00-18:00 IST, Website: www. Athrout.org/DocOnCall
- National Commission for Women (NCW) helpline: 0721-7735372
- Department of Social Welfare Jammu & Kashmir Integrated SAKHI – One Stop Centre Women Help Line – 181, Website: <http://181jandk.in/>

**BANGLADESH**

- Bangabandhu Sheikh Mujib Medical University (BSMMU), Shahbag, Dhaka-1000, +880255165760-94,: <https://bsmmu.edu.bd>
- National Institute of Mental Health, Dhaka, 261, Elephant Road, Dhaka-1205, <https://www.tuugo.com.bd/Companies/national-institute-of-mental-health-dhaka/16300065052>
- MONOSHEBA Online Mental Health App, <https://monosheba.com/about-us>
- BRAC Centre, 75 Mohakhali, Dhaka-1212. Tel: 880-2-9881265, Ext: 3161, 3182, 3191, E-mail: info@brac.net, Website: http://www.brac.net
- Acid Survivor Foundation, Plot # A/5, Block # A, CRP Building, 5th & 6th floor, Mirpur - 14, Dhaka – 1206. Hotlines: +880 9678777148, +880 9678777149, +88 01713010461. Email: asf@acidsurvivors.org, Website: https://acidsurvivors.org
- Ain o Salish Kendra (ASK), Address : 2/16, Block-B, Lalmatia, Dhaka-1207. Tel: 880-2-8100192, 8100195, 8100197, Mobile: 01714025069, Emergency contact : 01724415677 – 09:00 to 17:00, daily, E-mail: [ask@citechco.net](mailto:ask@citechco.net), Website: http://www.askbd.org
- BLAST, 1/1 Pioneer Road,Kakrail, Dhaka-1000, Tel: 0088-02-8391970-2, 8317185, Email: [mail@blast.org.bd](mailto:mail@blast.org.bd), Website: [www.blast.org.bd](http://www.blast.org.bd)
- Bangladesh National Woman Lawyers’ Association (BNWLA), Monico Mina Tower, West Agargaon, 48/3 Shahid Shahabuddin Shorok, Dhaka 1207, Tel: +8802-9143293, +88-02-9121925, E-mail: bnwlabjmas@gmail.com
- Amrai Pari (WE CAN) Secretariat, 6/4 A, Sir Sayed Road (2nd Floor), Mohammadpur, Dhaka-1207, Tel: +88 02 9130265, E-mail : info@wecan-bd.org, [wecan_secretariatbd@yahoo.com](mailto:wecan_secretariatbd@yahoo.com), Website: www.wecan-bd.org
- National Helpline Centre for Violence against Women and Children Department of Women Affairs (7th Floor), 37/3 Eskaton Garden Road, Dhaka-1000, Helpline number: 109, E-mail: [109helpline@gmail.com](mailto:109helpline@gmail.com), Website: www.nhc.gov.bd

**ZIMBABWE**

PHOEBE Zimbabwe, 136 Coronation Avenue Greendale,Harare, Zimbabwe, 00 263 242 492223. <http://phoebecentre.org.uk/zimbabwe/> operates women’s recovery colleges at:

- Harare Central Hospital, Talbot Road, Harare, Zimbabwe, 00 263 462 110019
- Annex Psychiatric Hospital, Cnr. Josiah Tongogara /Mazowe Street Parirenyatwa Annex, Harare, Zimbabwe

**UNITED KINGDOM**

- Samaritans <https://www.samaritans.org/> (freephone, 24 hours) 116 123
- <https://www.mind.org.uk/>
- Safe Lives <https://safelives.org.uk/>
- National domestic abuse helpline (freephone, 24 hours): 0808 2000 247 <https://www.nationaldahelpline.org.uk/>

In which country do you live? __________________

How old are you? ___________________

Are you currently in a relationship?

1. Yes
2. No

If you are currently in a relationship, how old is your partner? ______________

What is your gender?

1. Male
2. Female
3. Other: _________________

What is your ethnic group? Choose one option that best describes your ethnic group or background

White

1. English/Welsh/Scottish/Northern Irish/British
2. Irish
3. Gypsy or Irish Traveller
4. Any other White background, please describe

Mixed/Multiple ethnic groups

1. White and Black Caribbean
2. White and Black African
3. White and Asian
4. Any other Mixed/Multiple ethnic background, please describe

Asian/Asian British

9. Indian

10. Pakistani

11. Bangladeshi

12. Chinese

13. Any other Asian background, please describe

Black/ African/Caribbean/Black British

14. African

15. Caribbean

16. Any other Black/African/Caribbean background, please describe

Other ethnic group

17. Arab

18. Any other ethnic group, please describe

How many children do you have? _______________

Are you currently pregnant?

1. Yes
2. No

How many people live in your household?

Adults: _______________

Children: _____________

How many rooms are there in your home? _____________

How many adults live in your home? __________________

How many children live in your home? _________________

Has the number of people living in your home changed since COVID-19?

1. Yes – more people live in my home now
2. Yes – fewer people live in my home now
3. No change

What is your highest level of education?

1. Did not complete Primary School
2. Completed Primary School
3. Completed Secondary School
4. Undergraduate degree
5. Postgraduate degree
6. Unsure

What is your current occupation?

1. Student
2. Employed
3. Self-employed
4. Unemployed
5. Full-time carer of children or dependent adults
6. Other

We are interested in how your life has been affected by the coronavirus pandemic. Different places have taken different actions to reduce the spread of COVID-19, so we will ask you questions about whether things have changed since your country responded to COVID-19.

Has your occupation CHANGED since your country’s response to the coronavirus pandemic began?

1. Yes
2. No

If YES, please say how: _____________________

How has COVID-19 affected how much time you spend at home?

1. I spend more time at home than before
2. I spend less time at home than before
3. No change

How has COVID-19 affected how much time you spend with your partner?

1. I spend more time with my partner than before
2. I spend less time with my partner than before
3. No change
4. I’m not in a relationship

How has COVID-19 affected how much time you spend with adult family members other than your partner? These could be your parents, grandparents, aunts, uncles, cousins, nieces or nephews.

1. I spend more time with my family than before
2. I spend less time with my family than before
3. No change

How has COVID-19 affected how much time you spend with your children?

1. I spend more time with my children than before
2. I spend less time with my children than before
3. No change
4. I do not have children

How has your country’s response to the coronavirus pandemic affected your freedom to move around?

1. I stay at home all the time
2. I travel to just a few essential places
3. My movements have not changed

How has COVID-19 affected the way you spend your time? (Tick all that apply)

1. I spend more time on household chores (e.g. cleaning, washing) than before
2. I spend more time cooking than before
3. I spend more time doing things that I enjoy than before
4. I spend less time doing things that I enjoy than before
5. I spend more time working than before
6. I spend less time working than before

Do you have any reason to believe that you have had COVID-19?

1. Yes, I tested positive
2. Yes, I had symptoms, but I have not been tested
3. Yes, I had close contact with people who had COVID-19, but I have not been tested
4. No

Have you been unwell in the past 30 days? Please briefly summarise your illness and any treatment you received.

___________________________________________

**The following questions are related to certain pains and problems that may have bothered you in the last 30 days. If you had the problem in the last 30 days, please answer YES. If you did not have the problem in the last 30 days, answer NO.**

Do you often have headaches?

0 No

1 Yes

Is your appetite poor?

0 No

1 Yes

Do you sleep badly?

0 No

1 Yes

Are you easily frightened?

0 No

1 Yes

Do your hands shake?

0 No

1 Yes

Do you feel nervous, tense or worried?

0 No

1 Yes

Is your digestion poor?

0 No

1 Yes

Do you have trouble thinking clearly?

0 No

1 Yes

Do you feel unhappy?

0 No

1 Yes

Do you cry more than usual?

0 No

1 Yes

Do you find it difficult to enjoy your daily activities?

0 No

1 Yes

Do you find it difficult to make decisions?

0 No

1 Yes

Is your daily work suffering?

0 No

1 Yes

Are you unable to play a useful part in life?

0 No

1 Yes

Have you lost interest in things?

0 No

1 Yes

Do you feel that you are a worthless person?

0 No

1 Yes

Has the thought of ending your life been on your mind?

0 No

1 Yes

*Have you ever tried to end your life before?

0 No

1 Yes

Do you feel tired all the time?

0 No

1 Yes

Do you have uncomfortable feelings in your stomach?

0 No

1 Yes

Are you easily tired?

0 No

1 Yes

*Are other people getting on your nerves?

0 No

1 Yes

*Are you easily irritated?

0 No

1 Yes

*Have you felt angry?

0 No

1 Yes

*Have you been feeling like you might take your anger out on someone?

0 No

1 Yes

Have you been feeling like you might take your anger out on friends?

0 No

1 Yes

Have you been feeling like you might take your anger out on colleagues?

0 No

1 Yes

Have you been feeling like you might take your anger out on your partner or spouse?

0 No

1 Yes

Have you been feeling like you might take your anger out on family members?

0 No

1 Yes

Have you ever injured someone during a time when you felt angry?

0 No

1 Yes

Have you ever damaged property during a time when you felt angry?

0 No

1 Yes

Have your feelings of anger changed since COVID-19 started?

Yes – I feel angrier, or I feel angry more often

Yes – I feel less angry, or I feel angry less often

No – my feelings of anger have not changed

**COVID-19 changed many things about our work, our lives and our behaviours. These questions are about your life AT THE MOMENT and how it has changed since your country’s response to the coronavirus pandemic began. For example, many people drink alcohol or use recreational drugs during times of stress or anxiety.**

| **In your life, which of the following substances have you ever used?** | **No** | **Yes** |
| --- | --- | --- |
| Tobacco products (cigarettes, chewing tobacco, cigars, etc.) |  |  |
| Alcoholic beverages (beer, wine, spirits, etc.) |  |  |
| Cannabis (marijuana, pot, grass, hash, etc.) |  |  |
| Cocaine (coke, crack, etc.) |  |  |
| Amphetamine type stimulants (speed, diet pills, ecstasy, etc.) |  |  |
| Inhalants (nitrous, glue, petrol, paint thinner, etc.) |  |  |
| Sedatives or Sleeping Pills (Valium, Serepax, Rohypnol, etc.) |  |  |
| Sedatives or Sleeping Pills (Valium, Serepax, Rohypnol, etc.) |  |  |
| Hallucinogens (LSD, acid, mushrooms, PCP, Special K, etc.) |  |  |
| Opioids (heroin, morphine, methadone, codeine, etc.) |  |  |
| Other - specify: __________________ |  |  |

| **In the past three months, how often have you used the substances you mentioned?** | **Never** | **Once or twice** | **Monthly** | **Weekly** | **Daily or almost daily** |
| --- | --- | --- | --- | --- | --- |
| Tobacco products (cigarettes, chewing tobacco, cigars, etc.) |  |  |  |  |  |
| Alcoholic beverages (beer, wine, spirits, etc.) |  |  |  |  |  |
| Cannabis (marijuana, pot, grass, hash, etc.) |  |  |  |  |  |
| Cocaine (coke, crack, etc.) |  |  |  |  |  |
| Amphetamine type stimulants (speed, diet pills, ecstasy, etc.) |  |  |  |  |  |
| Inhalants (nitrous, glue, petrol, paint thinner, etc.) |  |  |  |  |  |
| Sedatives or Sleeping Pills (Valium, Serepax, Rohypnol, etc.) |  |  |  |  |  |
| Sedatives or Sleeping Pills (Valium, Serepax, Rohypnol, etc.) |  |  |  |  |  |
| Hallucinogens (LSD, acid, mushrooms, PCP, Special K, etc.) |  |  |  |  |  |
| Opioids (heroin, morphine, methadone, codeine, etc.) |  |  |  |  |  |
| Other - specify: __________________ |  |  |  |  |  |

| **Has your use of these substances CHANGED since your country’s response to the coronavirus pandemic began?** | **I’m using it more** | **I’m using it less** | **No change** |
| --- | --- | --- | --- |
| Tobacco products (cigarettes, chewing tobacco, cigars, etc.) |  |  |  |
| Alcoholic beverages (beer, wine, spirits, etc.) |  |  |  |
| Cannabis (marijuana, pot, grass, hash, etc.) |  |  |  |
| Cocaine (coke, crack, etc.) |  |  |  |
| Amphetamine type stimulants (speed, diet pills, ecstasy, etc.) |  |  |  |
| Inhalants (nitrous, glue, petrol, paint thinner, etc.) |  |  |  |
| Sedatives or Sleeping Pills (Valium, Serepax, Rohypnol, etc.) |  |  |  |
| Sedatives or Sleeping Pills (Valium, Serepax, Rohypnol, etc.) |  |  |  |
| Hallucinogens (LSD, acid, mushrooms, PCP, Special K, etc.) |  |  |  |
| Opioids (heroin, morphine, methadone, codeine, etc.) |  |  |  |
| Other - specify: __________________ |  |  |  |

**COVID-19 changed many things about our relationships with other people. These questions are about your relationships with other people AT THE MOMENT and how they have changed since your country’s response to the coronavirus pandemic began.**

Thinking about your relationships with your neighbours (people who live near your home), how many difficulties are you experiencing in your relationships with your neighbours?

1. Few difficulties
2. Some difficulties
3. Moderate difficulties
4. Severe difficulties

Has this changed since COVID-19 started?

1. Yes – difficulties with my neighbours have got worse
2. Yes – difficulties with my neighbours have got better
3. No change

How many difficulties are you experiencing in your relationships with friends (non-family members) at the moment?

1. Few difficulties
2. Some difficulties
3. Moderate difficulties
4. Severe difficulties

Has this changed since COVID-19 started?

1. Yes – my difficulties with my friends have got worse
2. Yes – my difficulties with my friends have got better
3. No change

How many difficulties are you experiencing in your relationship with your romantic or marital partner/spouse at the moment?

1. Few difficulties
2. Some difficulties
3. Moderate difficulties
4. Severe difficulties
5. I am not in a relationship with a partner at the moment

Has this changed since COVID-19 started?

1. Yes – my difficulties with my partner have got worse
2. Yes – my difficulties with my partner have got better
3. No change

How many difficulties are you experiencing in your relationships with other family members (non-partners/spouses)?

1. Few difficulties
2. Some difficulties
3. Moderate difficulties
4. Severe difficulties

Has this changed since COVID-19 started?

1. Yes – my difficulties with my other family members have got worse
2. Yes – my difficulties with my other family members have got better
3. No change

**COVID-19 changed many things about the communities where we live. These questions are about how safe you feel in different places AT THE MOMENT and how this has changed since your country’s response to the coronavirus pandemic began. By ‘safe’, we mean feeling that you are not likely to come to harm.**

How safe would you feel if you were walking alone outside your home during the day?

1. Very safe
2. Moderately safe
3. Neither safe nor unsafe
4. Moderately unsafe
5. Very unsafe

How has this changed since COVID-19 started?

1. I feel safer now
2. I feel less safe now
3. No change

How safe would you feel if you were walking alone outside your home at night time?

1. Very safe
2. Moderately safe
3. Neither safe nor unsafe
4. Moderately unsafe
5. Very unsafe

How has this changed since COVID-19 started?

1. I feel safer now
2. I feel less safe now
3. No change

How safe do you feel when you are inside your home during the day?

1. Very safe
2. Moderately safe
3. Neither safe nor unsafe
4. Moderately unsafe
5. Very unsafe

How has this changed since COVID-19 started?

1. I feel safer now
2. I feel less safe now
3. No change

How safe do you feel when you are inside your home at night time?

1. Very safe
2. Moderately safe
3. Neither safe nor unsafe
4. Moderately unsafe
5. Very unsafe

How has this changed since COVID-19 started?

1. I feel safer now
2. I feel less safe now
3. No change

How safe do you feel when you are at work (if you work outside your home)?

1. Very safe
2. Moderately safe
3. Neither safe nor unsafe
4. Moderately unsafe
5. Very unsafe
6. I do not work outside my home

How has this changed since COVID-19 started?

1. I feel safer now
2. I feel less safe now
3. No change

Do you feel that, at the moment, being at home is safer than being outside your home?

1. Yes
2. No

**How many people are so close to you that you can count on them if you have great personal problems?**

1. None
2. 1-2
3. 3-5
4. More than 5

How much interest and concern do people show in what you do?

1. None
2. Little
3. Uncertain
4. Some
5. A lot

How easy is it to get practical help from friends or neighbours if you should need it?

1. Very difficult
2. Difficult
3. Possible
4. Easy
5. Very easy

**Thank you for completing this survey. If you are experiencing difficulties, including stress, distress, physical or mental health problems, suicidal thoughts, abuse or violence, you are not alone. Your local health service can help. If you or someone else is at immediate risk, call emergency services for police or ambulance support. Below are some organisations who provide help, but if your country is not listed, you can find others online or by asking your health worker.**

**UGANDA**

- Butabika National Referral Mental Hospital <https://www.butabikahospital.go.ug/> (+256 414 504 376-day time +256 414 671 019 (Evening/Night Shift))
- Mental Health Uganda <https://www.mentalhealthuganda.org/> ([info@mentalhealthuganda.org](mailto:info@mentalhealthuganda.org) +256-392-178-953)
- Strong Minds <https://strongminds.org/> +256 200923340.
- Uganda Women’s Network <https://www.uwonet.or.ug/> (+256759330000)
- ALIGHT UGANDA PROGRAM <https://wearealight.org/> (toll free, 24 hours for refugees and asylum seekers +256771007447)

**ETHIOPIA**

- <http://www.epanets.org/>
- <https://www.womankind.org.uk/what-we-do/our-approach/partners/detail/association-for-women-s-sanctuary-and-development-(awsad)>

**ZAMBIA**

- PsycHealth Zambia https://psychzambia.com/ (+260955264975)
- Psychology Association of Zambia https://paz.co.zm/ (+260762024357)
- Strong Minds Zambia https://strongminds.org/ (+256 200923340)
- University Teaching Hospital, Clinic 6 /Department of Psychiatry https://www.unza.zm/schools/medicine/departments/psychiatry (+260211253947)
- Lifeline Zambia https://clzambia.org/ (933 Toll free) Victim Support Unit Desk available at every police station.

**INDIA**

- National Institute of Mental Health & Neurosciences (NIMHANS): 080 – 4611 0007 (24*7 Toll-Free Helpline)
- iCall Phonelines (Multilingual) +919152987821 (10 am to 8 pm, Monday to Saturday), chat: nULTA app (10:00-20:00, Monday to Saturday), email: [icall@tiss.edu](mailto:icall@tiss.edu)
- Dedicated COVID support helpline: +91 9152987820 (10:00-18:00 Monday to Saturday)
- Institute of Mental Health and Neurosciences, Kashmir (IMHANS-K) Tel: 0194-2410037, Covid-19 mental health helplines: 7006314064 or 6006175374
- IMHANS-K in collaboration with Athrout COVID 19 dedicated mental health helplines: 6006613436, 6006613437 or 6006613438 10:00-13:00 and 16:00-18:00 IST, Website: www. Athrout.org/DocOnCall
- National Commission for Women (NCW) helpline: 0721-7735372
- Department of Social Welfare Jammu & Kashmir Integrated SAKHI – One Stop Centre Women Help Line – 181, Website: <http://181jandk.in/>

**BANGLADESH**

- Bangabandhu Sheikh Mujib Medical University (BSMMU), Shahbag, Dhaka-1000, +880255165760-94,: <https://bsmmu.edu.bd>
- National Institute of Mental Health, Dhaka, 261, Elephant Road, Dhaka-1205, <https://www.tuugo.com.bd/Companies/national-institute-of-mental-health-dhaka/16300065052>
- MONOSHEBA Online Mental Health App, <https://monosheba.com/about-us>
- BRAC Centre, 75 Mohakhali, Dhaka-1212. Tel: 880-2-9881265, Ext: 3161, 3182, 3191, E-mail: info@brac.net, Website: http://www.brac.net
- Acid Survivor Foundation, Plot # A/5, Block # A, CRP Building, 5th & 6th floor, Mirpur - 14, Dhaka – 1206. Hotlines: +880 9678777148, +880 9678777149, +88 01713010461. Email: asf@acidsurvivors.org, Website: https://acidsurvivors.org
- Ain o Salish Kendra (ASK), Address : 2/16, Block-B, Lalmatia, Dhaka-1207. Tel: 880-2-8100192, 8100195, 8100197, Mobile: 01714025069, Emergency contact : 01724415677 – 09:00 to 17:00, daily, E-mail: [ask@citechco.net](mailto:ask@citechco.net), Website: http://www.askbd.org
- BLAST, 1/1 Pioneer Road,Kakrail, Dhaka-1000, Tel: 0088-02-8391970-2, 8317185, Email: [mail@blast.org.bd](mailto:mail@blast.org.bd), Website: [www.blast.org.bd](http://www.blast.org.bd)
- Bangladesh National Woman Lawyers’ Association (BNWLA), Monico Mina Tower, West Agargaon, 48/3 Shahid Shahabuddin Shorok, Dhaka 1207, Tel: +8802-9143293, +88-02-9121925, E-mail: bnwlabjmas@gmail.com
- Amrai Pari (WE CAN) Secretariat, 6/4 A, Sir Sayed Road (2nd Floor), Mohammadpur, Dhaka-1207, Tel: +88 02 9130265, E-mail : info@wecan-bd.org, [wecan_secretariatbd@yahoo.com](mailto:wecan_secretariatbd@yahoo.com), Website: www.wecan-bd.org
- National Helpline Centre for Violence against Women and Children Department of Women Affairs (7th Floor), 37/3 Eskaton Garden Road, Dhaka-1000, Helpline number: 109, E-mail: [109helpline@gmail.com](mailto:109helpline@gmail.com), Website: www.nhc.gov.bd

**ZIMBABWE**

PHOEBE Zimbabwe, 136 Coronation Avenue Greendale,Harare, Zimbabwe, 00 263 242 492223. <http://phoebecentre.org.uk/zimbabwe/> operates women’s recovery colleges at:

- Harare Central Hospital, Talbot Road, Harare, Zimbabwe, 00 263 462 110019
- Annex Psychiatric Hospital, Cnr. Josiah Tongogara /Mazowe Street Parirenyatwa Annex, Harare, Zimbabwe

**UNITED KINGDOM**

- Samaritans <https://www.samaritans.org/> (freephone, 24 hours) 116 123
- <https://www.mind.org.uk/>
- Safe Lives <https://safelives.org.uk/>
- National domestic abuse helpline (freephone, 24 hours): 0808 2000 247 <https://www.nationaldahelpline.org.uk/>
